# Mitophagy–pyroptosis–macrophage polarization crosstalk in cardiovascular diseases: mechanistic insights and therapeutic potential

**DOI:** 10.3389/fcvm.2026.1886093

**Published:** 2026-09-02

**Authors:** Jieying Yuan, Tingru Ji, Ruquan Chen, Yuesong Yuan, Lin Ji

**Affiliations:** 1 College of Acupuncture and Massage, Shandong University of Traditional Chinese Medicine, Jinan, Shandong, China; 2 College of International Education College, Shandong University of Traditional Chinese Medicine, Jinan, Shandong, China; 3 Medical Services Department, Second Affiliated Hospital of Shandong University of Traditional Chinese Medicine, Jinan, Shandong, China

**Keywords:** cardiovascular disease, immunometabolic regulation, macrophage polarization, mitochondrial quality control, mitophagy, pyroptosis

## Abstract

Cardiovascular disease (CVD) is one of the leading causes of global morbidity and mortality. Its development and progression are closely associated with mitochondrial dysfunction, sterile inflammation, and immunometabolic dysregulation. Mitophagy, a key mechanism of mitochondrial quality control, maintains mitochondrial homeostasis by selectively removing damaged mitochondria. However, either insufficient or excessive mitophagy may disrupt cellular metabolism, promote ROS production and mitochondrial DNA (mtDNA) release, and activate inflammatory signaling pathways, thereby aggravating cardiovascular injury. Increasing evidence indicates that mitophagy is closely linked to pyroptosis and macrophage polarization. Impaired mitophagy can enhance inflammasome activation and gasdermin-mediated pyroptosis. In turn, inflammatory mediators released during pyroptosis may further impair mitochondrial quality control, forming a self-amplifying inflammatory loop. Meanwhile, mitophagy regulates macrophage metabolic reprogramming and phenotypic switching, thereby influencing the balance between pro-inflammatory M1-like responses and reparative M2-like functions. This review summarizes the molecular mechanisms underlying the crosstalk among mitophagy, pyroptosis, and macrophage polarization in CVD, with particular emphasis on myocardial infarction (MI) and myocardial ischemia-reperfusion injury. Current evidence suggests that restoring appropriate mitophagic flux, inhibiting aberrant pyroptosis, and reshaping macrophage phenotypes may help alleviate inflammatory injury and adverse cardiac remodeling. This review aims to provide a mechanistic framework for immunometabolic regulation in CVD and to support the development of precision therapeutic strategies targeting mitochondrial quality control and inflammatory cell responses.

## 1 Introduction

Cardiovascular disease (CVD) remains a major global health burden. According to the World Health Organization, approximately 19.8 million deaths were attributed to CVD in 2022, accounting for 32% of all global deaths. Ischemic heart disease and stroke were the leading causes of CVD-related death, and more than three-quarters of these deaths occurred in low- and middle-income countries. In parallel, the clinical burden of ischemic heart disease, heart failure (HF), and atherosclerosis (AS) continues to increase (1).

The pathophysiology of CVD is complex. Aseptic inflammation and immunometabolic dysregulation have been recognized as key contributors to cardiovascular structural remodeling and functional impairment (2–4). The heart has a high energy demand, and the maintenance of its structure and function depends largely on mitochondrial quantity and functional integrity. Mitophagy, a key component of mitochondrial quality control, selectively recognizes and removes damaged or aged mitochondria through autophagosome- and lysosome-dependent pathways (5, 6). Under physiological conditions, mitophagy helps maintain mitochondrial network stability and cellular energy metabolism. However, under pathological stress, including ischemia, hypoxia, and lipid metabolic disorders, autophagic flux is often impaired. This impairment promotes the cytoplasmic accumulation of dysfunctional mitochondria, leading to mitochondrial homeostatic imbalance and myocardial tissue injury. Such alterations have been observed in several cardiovascular phenotypes, including AS, myocardial ischemia-reperfusion injury (MIRI), HF, and cardiomyopathy (7, 8).

Current evidence suggests that abnormal mitophagy disrupts metabolic homeostasis in cardiac and vascular cells. It also reshapes the local inflammatory microenvironment by promoting reactive oxygen species (ROS) accumulation, mitochondrial DNA (mtDNA) release, and inflammasome activation. In addition, abnormal mitophagy is closely associated with pyroptosis and macrophage polarization (9, 10).

However, most existing reviews on CVD have focused on mitophagy, pyroptosis, or macrophage polarization as separate topics, with limited systematic discussion of the interactions between mitophagy and pyroptotic signaling pathways and their roles in macrophage functional remodeling (11, 12). Therefore, this review adopts mitophagy as the central framework to investigate how mitochondrial quality control imbalance contributes to cardiovascular inflammation and injury through the activation of pyroptosis and the regulation of macrophage polarization. The major regulatory mechanisms of mitophagy are first introduced, followed by an analysis of its crosstalk with pyroptotic pathways and its impact on macrophage polarization through the regulation of mitochondrial homeostasis and inflammatory microenvironment remodeling. By integrating these mechanisms, this review aims to provide a deeper understanding of the role of mitophagy in cardiovascular inflammatory regulation and establish a theoretical basis for therapeutic strategies targeting mitochondrial quality control.

## 2 Molecular mechanisms of mitophagy

Since Lemasters and colleagues first proposed the concept of “mitochondrial autophagy” in 2005, the molecular mechanisms of this process have attracted increasing attention (13). Mitophagy is a form of selective autophagy that depends on coordinated interactions between autophagosomes and lysosomes (14). Based on the requirement for ubiquitination, mitophagy can be broadly classified into ubiquitin-dependent and receptor-mediated, ubiquitin-independent pathways.

The ubiquitin-dependent pathway is one of the best-characterized mechanisms of mitophagy. In mammals, the PTEN-induced kinase 1 (PINK1)/Parkin pathway is the most representative example (15, 16). Under physiological conditions, PINK1 is imported into mitochondria and cleaved after reaching the inner mitochondrial membrane. However, when mitochondrial membrane potential is disrupted, PINK1 import and cleavage are impaired. As a result, PINK1 accumulates on the outer mitochondrial membrane (OMM) and undergoes autophosphorylation, which promotes Parkin recruitment and activation (17, 18). Activated Parkin functions as an E3 ubiquitin ligase and ubiquitinates OMM proteins to generate polyubiquitin chains. These chains are then recognized by autophagy receptors, including p62 and optineurin (OPTN). This recognition promotes autophagosome formation around damaged mitochondria and facilitates their lysosomal degradation (15, 19).

Receptor-mediated, ubiquitin-independent mitophagy is mainly driven by mitophagy receptors located on the OMM. These receptors directly recognize mitochondrial cargo and recruit the autophagic machinery. Key molecules include BNIP3, BNIP3-like protein (BNIP3L, also known as NIX), FUN14 domain-containing protein 1 (FUNDC1), FKBP prolyl isomerase 8 (FKBP8), and BCL2-like protein 13 (BCL2L13) (20–23). Unlike the PINK1/Parkin pathway, this mechanism does not require extensive ubiquitination of OMM proteins. Instead, these receptors use their LC3-interacting region (LIR) motifs to bind LC3/GABARAP proteins directly, thereby anchoring damaged mitochondria to the expanding isolation membrane (24–26). Receptor-mediated mitophagy pathways may operate under basal and stress conditions. These mechanisms may also interact in complementary or compensatory ways, particularly when ubiquitination signals are weak or PINK1/Parkin activity is limited (27).

Appropriate mitophagy protects cells by maintaining mitochondrial quality control. However, in CVD-related injury, defective or excessive mitophagy may contribute to maladaptive cellular damage (28). Insufficient mitophagy promotes the accumulation of damaged mitochondria, aggravates oxidative stress, disrupts cellular structural integrity, and impairs cellular function (29). Conversely, excessive mitophagy may remove functional mitochondria or activate cell death-related signaling pathways. Therefore, an imbalance in mitophagy is closely associated with HF, MIRI, AS, cardiomyopathy, and cardiac hypertrophy (30). Related therapeutic strategies are summarized in Table 1.

**Table 1:** Translational grading of therapeutic strategies targeting mitophagy-related cardiovascular crosstalk.

Evidence level	Intervention or strategy	Model/system	Major target cell(s)	Principal findings and mechanism	Major translational limitation
Clinical trial (91)	Empagliflozin	HFrEF randomized clinical trial (EMPEROR-Reduced)	Not assessed at cellular level	Reduced cardiovascular death or HF hospitalization; mitophagy and macrophage mechanisms remain unvalidated clinically	Crosstalk mechanism not assessed in patients
Clinical trial (94)	Semaglutide	SELECT trial in obesity with established CVD	Not assessed at cellular level	Reduced major cardiovascular events; mechanistic links to mitophagy or pyroptosis remain experimental	Crosstalk mechanism not clinically validated
Human mechanistic study (93)	Empagliflozin	T2DM patients after MI; patient-derived monocyte macrophages	Monocyte-derived macrophages	Reduced IL1B transcription, ATP release, and caspase-1 activity after inflammatory stimulation	Mitophagic flux not directly assessed
Human pilot trial (97)	MitoQ	Randomized crossover trial in healthy older adults	Vascular endothelium	Improved endothelial function and reduced oxidative stress-related impairment	Small non-CVD cohort; no cardiovascular outcome assessment
Animal/cell (58)	VX765	ApoE−/− and Ldlr−/− mice; macrophage models	Macrophages	Promoted Parkin recruitment and mitophagy, suppressed NLRP3/caspase-1/GSDMD pyroptosis, and improved efferocytosis	Preclinical evidence only
Cell model (59)	Hirudin	Ang II-induced H9c2 cardiomyocyte hypertrophy model	Cardiomyocytes	Activated PINK1/Parkin mitophagy and reduced ROS and NLRP3 inflammasome activation	Limited to an *in vitro* model
Animal/cell (60)	SIRT6-enriched ASC exosomes	MIRI cell and animal models	Cardiomyocytes	Enhanced mitophagy-related signaling and inhibited AIM2/GSDMD pyroptosis	Clinical safety and delivery remain unvalidated
Animal/cell (49)	AIBP	Atherosclerosis mouse models and macrophage systems	Macrophages	Regulated PINK1-dependent mitophagy and macrophage inflammatory polarization	Mainly preclinical/ genetic evidence
Animal/cell (95)	miR-21-3p inhibitor	HF animal model and macrophage–cardiomyocyte coculture	Cardiomyocytes/macrophages	Reduced excessive mitophagy-associated injury and modulated macrophage polarization	Clinical delivery and efficacy remain unclear
Animal/cell (86)	Empagliflozin	Cardiac microvascular I/R models	Endothelial cells	Activated AMPK*α*1/ULK1/FUNDC1-mediated mitophagy and improved endothelial function	Macrophage crosstalk not directly assessed
Cell model (57)	Semaglutide	H/R cardiomyocyte model	Cardiomyocytes	Enhanced PINK1/Parkin-related mitophagy signaling and reduced oxidative stress	Limited to a cellular model
Animal/cell (96)	MitoQ	Diabetic MIRI models	Cardiomyocytes	Enhanced PINK1/Parkin-associated mitophagy and reduced oxidative injury	Crosstalk mechanism not directly assessed
Animal/cell (98)	Resveratrol	MI models	Macrophages	Modulated inflammatory macrophage polarization and reparative responses	Mitophagy not directly evaluated
Animal/cell (99)	Curcumin	MI models	Macrophages	Reduced inflammatory macrophage phenotype and improved cardiac remodeling	Mitophagy not directly evaluated
Animal model (100)	Melatonin	Myocardial I/R model	Cardiac tissue/cardiomyocytes	Regulated excessive mitophagy through Apelin/SIRT3 signaling	Macrophage responses not directly assessed
Animal/cell (101)	Empagliflozin-pretreated BMSC exosomes	Cardiomyocyte and MIRI models	Cardiomyocytes	Activated ATAD3A/PINK1/Parkin-related mitophagy and improved cardiac injury	Delivery, standardization, and long-term safety remain unclear

## 3 Crosstalk between mitophagy and pyroptosis

### 3.1 Basic biology of pyroptosis

Pyroptosis is an inflammatory form of programmed cell death mediated by inflammasomes and inflammatory caspases (31). It is characterized by cell swelling, membrane pore formation, plasma membrane rupture, and the release of intracellular contents, which further amplifies inflammation (32, 33).

During pyroptosis, pathogen-associated molecular patterns (PAMPs) or damage-associated molecular patterns (DAMPs) promote the assembly of inflammasomes, particularly the NOD-like receptor family pyrin domain-containing 3 (NLRP3) inflammasome. The activated NLRP3 inflammasome then activates caspase-1, which cleaves gasdermin D (GSDMD) (34–36). The N-terminal fragment of cleaved GSDMD oligomerizes and forms pores in the plasma membrane. These pores induce cell swelling and eventually lead to membrane rupture (37). At the same time, caspase-1 promotes the maturation of interleukin-1*β* (IL-1*β*) and interleukin-18 (IL-18). These inflammatory cytokines are then released through GSDMD-mediated pores into the extracellular space, thereby intensifying the local inflammatory response (38).

Current evidence indicates that mitophagy and pyroptosis regulate each other in cardiovascular-related diseases (11, 12). Appropriate mitophagy removes damaged mitochondria and reduces the release of ROS and mitochondrial damage-associated molecular patterns (mtDAMPs), including mtDNA. This process limits inflammasome assembly and suppresses subsequent pyroptosis (39–41). In contrast, under pathological conditions, impaired mitophagic flux promotes the accumulation of damaged mitochondria, which can activate downstream pyroptotic signaling cascades (42, 43). Meanwhile, inflammatory mediators and intracellular contents released during pyroptosis further amplify local inflammation, aggravate mitochondrial damage, and impair mitochondrial quality control. These events may form a self-reinforcing cycle involving mitochondrial damage, inflammasome activation, pyroptosis, and inflammatory amplification, ultimately contributing to the progression of cardiovascular-related diseases (44–46).

### 3.2 Molecular crosstalk between mitophagy and pyroptosis in CVD

Ubiquitin-dependent mitophagy plays a critical regulatory role in the interaction between mitophagy and pyroptosis. However, the consequences of this interplay vary among different cardiovascular cell populations (11, 12). In cardiomyocytes, impaired mitophagy primarily contributes to energy dysfunction, oxidative stress, and inflammatory cell death, ultimately leading to cardiac dysfunction (47). In endothelial cells, mitophagy dysregulation may disrupt vascular homeostasis and exacerbate inflammatory responses (48). In macrophages, mitochondrial quality control not only regulates inflammasome activation but also participates in metabolic reprogramming and the transition of inflammatory phenotypes (49). Ubiquitin-specific peptidase 30 (USP30) is a deubiquitinating enzyme located on the outer mitochondrial membrane. Studies have shown that impaired mitophagy flux is associated with elevated USP30 expression in HF mouse models and oxygen-glucose deprivation (OGD)-treated H9C2 cardiomyocyte models (47). USP30 inhibition activates the PINK1/Parkin pathway and reduces the expression of NLRP3, caspase-1, GSDMD-N, IL-1*β*, and IL-18. These effects alleviate cardiomyocyte pyroptosis and improve cardiac function (47). Recent studies have further shown that, in myocardial infarction (MI) mouse models and OGD-induced H9C2 cardiomyocyte models, the environmental pollutant benzo[a]pyrene (3,4-B[a]P) suppresses PINK1/Parkin-dependent mitophagy through the aryl hydrocarbon receptor pathway (50). This inhibition induces abnormal opening of the mitochondrial permeability transition pore (mPTP), enhances NLRP3 inflammasome activation, and promotes pyroptosis, thereby aggravating myocardial injury (50).

In an MI model, triggering receptor expressed on myeloid cells 1 (TREM1) expression is upregulated in macrophages. Studies using macrophage-specific models have shown that TREM1 suppresses PINK1/Parkin-mediated mitophagy and promotes NLRP3-dependent pyroptosis, thereby amplifying inflammatory responses and exacerbating myocardial injury (51). Further investigations demonstrated that macrophage-specific TREM1 knockdown restores mitophagy activity, inhibits pyroptotic signaling activation, and improves cardiac function while reducing myocardial fibrosis and adverse cardiac remodeling (51).

Beyond the classical PINK1/Parkin-mediated ubiquitin-dependent mitophagy pathway, receptor-mediated ubiquitin-independent mitophagy also contributes to the regulation of mitochondrial homeostasis and pyroptotic responses in macrophages. Reduced NIX expression has been observed in vascular tissues from patients with AS (52). Further studies in oxidized low-density lipoprotein (ox-LDL)-stimulated macrophage models demonstrated that NIX silencing decreases mitophagy activity, accompanied by ROS accumulation, NLRP3 inflammasome activation, increased caspase-1 activation, and enhanced IL-1*β* maturation, thereby promoting macrophage pyroptosis (52). However, it is important to note that cardiac macrophages exhibit substantial heterogeneity. Resident cardiac macrophages and recruited monocyte-derived macrophages differ in their origins, metabolic states, and inflammatory functions. Currently, comparative studies investigating the regulatory mechanisms of TREM1- and NIX-mediated mitophagy–pyroptosis interactions among distinct macrophage subsets remain limited.

Similarly, in mouse models of MIRI and corresponding H9C2 cardiomyocyte models, upregulation of sirtuin 3 (SIRT3) and BNIP3 promotes mitophagy, suppresses GSDME cleavage and pyroptosis, and is associated with reduced infarct size (53). Epigenomic evidence further supports this view. In MIRI mouse and cardiomyocyte models, methyltransferase-like 3 (METTL3) maintains high early growth response 1 (EGR1) expression through N6-methyladenosine (m6A) modification and inhibits the Janus kinase 2/signal transducer and activator of transcription 3 (JAK2/STAT3) pathway. These changes aggravate mitochondrial dysfunction and impaired mitophagy, ultimately inducing GSDMD-dependent cardiomyocyte pyroptosis and promoting MIRI progression (54). Together, these findings indicate that impaired mitophagy, regardless of the specific pathway involved, may exacerbate myocardial injury by promoting inflammasome assembly and gasdermin cleavage, thereby enhancing pyroptosis.

**Figure 1:**
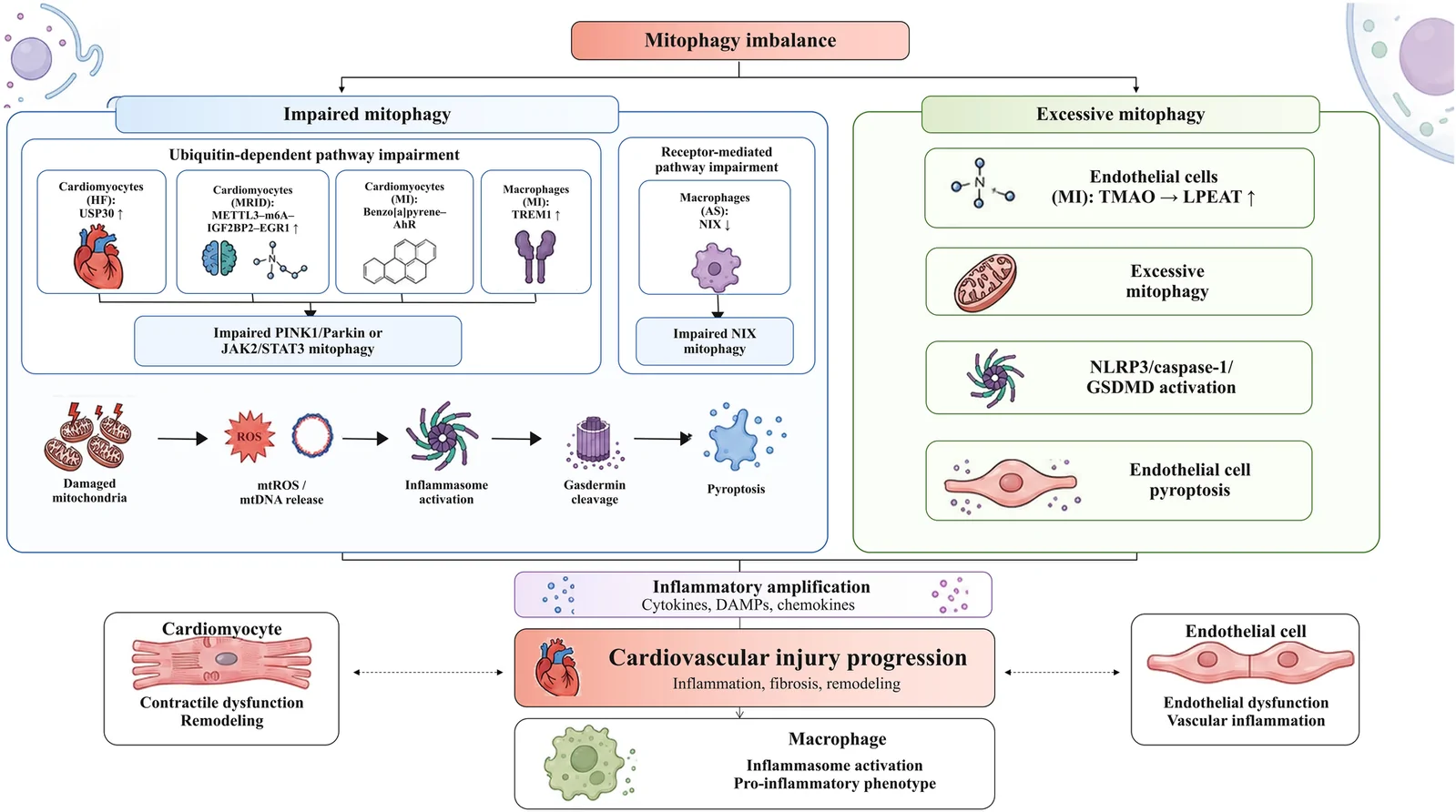
Cell type–specific crosstalk between mitophagy imbalance and pyroptosis in cardiovascular diseases.

Mitophagy appears to have an optimal regulatory range. Both insufficient and excessive mitophagy may induce pathological effects and promote tissue injury. Therefore, excessive mitophagy may also participate in pyroptosis regulation, in addition to the mechanism by which impaired mitophagy activates pyroptosis. For example, trimethylamine-N-oxide (TMAO) induces excessive mitophagy in vascular endothelial cells by upregulating lysophosphatidylethanolamine acyltransferase (LPEAT). It also activates the NLRP3/caspase-1/GSDMD pathway (55). Further details are provided in Figure 1.

### 3.3 Therapeutic potential of interventions targeting mitophagy–pyroptosis crosstalk in CVD

Given the important role of mitophagy imbalance and pyroptosis activation in the progression of CVDs, therapeutic strategies targeting this regulator*y* axis have attracted increasing attention. However, the translational evidence supporting different intervention approaches varies considerably. Some drugs have demonstrated cardiovascular benefits in clinical studies, although whether these effects are mediated through the mitophagy–pyroptosis axis remains largely supported by preclinical evidence. In contrast, strategies that directly modulate mitochondrial quality control or inflammatory cell death are still primarily at the cellular and animal experimental stages.

Glucagon-like peptide-1 receptor agonists (GLP-1RAs) represent a class of cardiometabolic agents with relatively robust clinical evidence and have demonstrated cardiovascular protective effects in patients with HF, diabetes, and CKD (56). Preclinical studies suggest that GLP-1RAs may promote mitophagy and improve autophagic flux through the regulation of signaling pathways such as SIRT1/Parkin and AMPK–mTOR, while reducing ROS production and the expression of inflammatory mediators, including interleukin-6 (IL-6), IL-1*β*, and tumor necrosis factor-α (TNF-α) (56, 57). These effects may contribute to their protective roles in models of MIRI, post-MI remodeling, and metabolically induced cardiovascular injury (56). However, it should be emphasized that current clinical benefits cannot be directly attributed to modulation of the mitophagy–pyroptosis axis, and further clinical and translational studies are required to validate this mechanism.

In contrast, several intervention strategies targeting the mitophagy–pyroptosis crosstalk have obtained mechanistic evidence from animal and cellular models. For example, the caspase-1 inhibitor VX765 primarily acts on macrophages in AS models. VX765 suppresses caspase-1 activation, thereby reducing NLRP3 inflammasome-mediated inflammatory responses, while promoting Parkin recruitment and mitophagy. This improves macrophage phagocytic clearance capacity and facilitates M2-like polarization, ultimately alleviating vascular inflammation and AS progression (58).

Furthermore, several strategies targeting mitochondrial homeostasis in cardiomyocytes have shown potential therapeutic value. Hirudin, for instance, activates PINK1/Parkin-mediated mitophagy in an Ang II-induced H9C2 cardiomyocyte hypertrophy model, reducing ROS accumulation and inhibiting NLRP3 inflammasome-associated pyroptotic signaling, thereby attenuating hypertrophic cardiac injury (59). Similarly, sirtuin 6 (SIRT6)-enriched adipose-derived stem cell exosomes have been reported to enhance mitophagy-related signaling and suppress absent in melanoma 2 (AIM2)/GSDMD-mediated pyroptosis in MIRI models, leading to improved cardiac function and reduced tissue injury (60). This study, which was primarily based on cardiomyocyte models and animal experiments, provides a potential direction for developing delivery strategies targeting mitochondrial quality control (60).

Overall, intervention strategies targeting the mitophagy–pyroptosis axis have demonstrated potential therapeutic effects in various CVD models. However, current evidence is still largely derived from cellular and animal studies, and their clinical efficacy, safety, and applicable populations remain insufficiently validated. Future studies should integrate clinical investigations, single-cell technologies, and precision delivery approaches to further define the therapeutic window of the mitophagy–pyroptosis regulatory network across different cell types and disease stages.

## 4 Mitophagy and macrophage polarization

### 4.1 Basic biology of macrophage polarization

Macrophages are key components of the innate immune system, and their phagocytic function was first systematically characterized through the pioneering work of Ilya Metchnikoff (61). Beyond phagocytosis, macrophages mediate antigen presentation, remove apoptotic cells, regulate inflammatory responses, and contribute to tissue repair. In response to signals within the local microenvironment, they can adopt distinct functional states that influence the initiation, progression, and resolution of inflammation. Macrophages were traditionally considered to arise predominantly from bone marrow-derived monocytes that enter tissues through the circulation and subsequently differentiate into mature macrophages (62, 63). However, recent evidence indicates that many tissue-resident macrophage populations originate from embryonic progenitors in the yolk sac or fetal liver and can persist through local self-renewal (64). Their developmental origins and tissue-specific niches contribute to substantial phenotypic and functional heterogeneity. For example, microglia, Kupffer cells, and osteoclasts are specialized macrophage-lineage cells with distinct roles in the central nervous system, liver, and bone, respectively (65, 66). Macrophage polarization refers to the acquisition of context-dependent functional states in response to spatially and temporally regulated environmental cues (67). In the classical framework, M1-like macrophages are induced by stimuli such as lipopolysaccharide (LPS), interferon-*γ* (IFN-*γ*), and tumor necrosis factor-α (TNF-α). These cells express inducible nitric oxide synthase (iNOS), generate nitric oxide (NO) and ROS, and release pro-inflammatory mediators, including TNF-α, IL-1*β*, and IL-6. These activities contribute to antimicrobial defense and inflammatory tissue responses (62). By contrast, M2-like activation is commonly associated with cytokines such as IL-4 and IL-13, whereas IL-10 and transforming growth factor-*β* (TGF-*β*) promote related regulatory and inflammation-resolving phenotypes. These macrophages frequently exhibit increased arginase-1 (Arg-1) expression and enhanced production of anti-inflammatory or reparative mediators. Accordingly, they participate in efferocytosis, inflammation resolution, angiogenesis, extracellular matrix remodeling, and tissue repair (68, 69). Nevertheless, the M1/M2 framework represents a simplified experimental model rather than a complete description of macrophage biology. *In vivo* macrophage phenotypes are highly plastic and distributed across a multidimensional continuum. They may also display context-specific states, including those described as Mox, M4, and M (Hb) macrophages (70, 71). During inflammatory responses, macrophage populations may initially acquire predominantly pro-inflammatory and antimicrobial functions. As inflammation resolves, local signals can promote the emergence of regulatory and reparative phenotypes that facilitate apoptotic-cell clearance, tissue reconstruction, and the restoration of homeostasis (72, 73). This transition is not strictly linear or uniform, because multiple macrophage states may coexist and dynamically change according to disease stage, tissue context, and environmental stimuli.

Macrophages exhibit marked temporal and spatial heterogeneity after MI. By integrating single-cell RNA sequencing with spatial transcriptomics, Jung and colleagues (74) identified inflammatory macrophage states as a prominent feature of the early infarct phase. These states were subsequently accompanied by the emergence of TREM2-high macrophages associated with lipid metabolism, efferocytosis, and tissue repair. Rizzo et al. reported similar findings in an independent experimental model. They further suggested that efferocytosis may promote the acquisition of reparative macrophage features (75). Accordingly, this review describes macrophage populations according to their ontogeny, differentiation state, metabolic profile, and functional properties. The terms “M1” and “M2” are retained only when discussing studies that explicitly use classical *in vitro* polarization protocols.

### 4.2 Crosstalk between mitophagy and macrophage polarization in CVD

The pathobiology of MI and MIRI extends beyond hypoxic cardiomyocyte death. It also involves profound immune remodeling and metabolic dysfunction (76, 77). Macrophage functional reprogramming and mitophagy are closely interconnected processes that influence inflammatory persistence, tissue repair, and ventricular remodeling (78, 79). However, their relationship cannot be reduced to the simple assumption that enhanced mitophagy invariably promotes M2 polarization. Instead, this crosstalk depends on mitochondrial quality control within macrophages, damage-associated signals released by cardiovascular cells, and reciprocal communication between macrophages and neighboring cells. Its biological effects also vary according to cell type, disease stage, tissue niche, and mitophagic flux.

After MI, the early inflammatory phase is characterized by the recruitment of CCR2-positive monocytes and the accumulation of monocyte-derived macrophages. These cells produce pro-inflammatory mediators, including tumor necrosis factor-α (TNF-α), IL-1*β*, and IL-6, while removing necrotic tissue. During the subsequent reparative phase, the composition and functional states of the macrophage population change. Reparative macrophage programs support efferocytosis, angiogenesis, fibroblast regulation, extracellular matrix deposition, and scar maturation (80). This process is not a uniform or strictly linear transition from M1 to M2 macrophages. Rather, multiple inflammatory and reparative states coexist and change dynamically throughout infarct healing.

Mitochondrial injury is a major link between ischemic damage and macrophage activation. Ischemia, hypoxia, and reperfusion increase mitochondrial reactive oxygen species (mtROS), reduce mitochondrial membrane potential, and promote the release of mtDNA. These changes can engage mitophagy pathways mediated by PINK1/Parkin, BCL2-interacting protein 3 (BNIP3)/NIX, and FUNDC1 (2). Nevertheless, mitochondrial stress does not necessarily result in effective mitochondrial clearance. Severe or persistent injury may impair autophagic flux, leading to the accumulation of dysfunctional mitochondria. Thus, the biological consequences of mitophagy depend on both its activation and the completion of mitochondrial degradation.

When mitophagy is impaired, damaged mitochondria continue to generate mtROS and release mtDNA. These mitochondrial danger signals can activate inflammasomes and cytosolic DNA-sensing pathways, thereby sustaining pro-inflammatory macrophage programs. By contrast, intact mitophagic flux limits the accumulation of mitochondrial damage and supports lysosomal function, efferocytosis, and metabolic homeostasis. Choi et al. reported that apolipoprotein A-I–binding protein (AIBP) supports mitochondrial quality control in macrophages exposed to oxidized low-density lipoprotein (81). Duan and colleagues further showed that hematopoietic AIBP deficiency in low-density lipoprotein receptor-deficient mice attenuated PINK1-dependent mitophagy, prolonged pro-inflammatory macrophage activation, and accelerated atherosclerosis (49). These findings support a role for macrophage mitophagy in limiting chronic vascular inflammation.

Mitophagy can regulate macrophage function through at least three interconnected mechanisms. First, PINK1/Parkin- or BNIP3/NIX-mediated clearance of damaged mitochondria reduces mtROS production and limits oxidative amplification of inflammatory signaling (7). Second, mitophagy restrains inflammasome activation. For example, APPL1–Rab5-mediated enhancement of mitophagy suppresses NLRP3 inflammasome activation and reduces IL-1*β* release (82). Third, mitochondrial quality control contributes to immunometabolic reprogramming. Effective mitophagy helps preserve oxidative phosphorylation (OXPHOS), fatty acid oxidation, and lysosomal function. These metabolic adaptations can support efferocytosis and selected IL-10- and TGF-*β* reparative programs (83). Therefore, mitophagy does not directly impose a fixed M2 phenotype. Instead, it creates a metabolic and redox environment that can favor specific reparative functions.

Macrophage activation states can also influence mitochondrial integrity and mitophagic flux. Pro-inflammatory signaling through Toll-like receptor 4, IFN-*γ*, TNF-α, and iNOS can disrupt electron transport, impair mitochondrial respiration, and promote mitochondrial depolarization. Persistent inflammatory activation may therefore increase the mitochondrial damage burden and compromise mitochondrial quality control (81). In an experimental MI model, myeloid deletion of the mitochondrial complex I subunit Ndufs4 caused sustained pro-inflammatory macrophage activation, impaired efferocytosis, delayed inflammation resolution, and worsened post-infarction repair (27). These findings indicate that inflammatory activation and mitochondrial dysfunction can reinforce each other. Conversely, signaling through IL-4/signal transducer and activator of transcription 6, peroxisome proliferator-activated receptor-*γ*/peroxisome proliferator-activated receptor-*γ* coactivator-1*β*, and IL-10/signal transducer and activator of transcription 3 supports oxidative metabolism and mitochondrial renewal. These pathways may thereby stabilize selected reparative macrophage programs (84).

The crosstalk between mitophagy and macrophage function has been most extensively investigated in MI, myocardial I/R injury, and atherosclerosis. Cardiomyocytes are an important source of mitochondrial damage-associated signals after I/R. In mice with cardiomyocyte-specific PARKIN overexpression, Li and colleagues found that enhanced clearance of damaged mitochondria reduced cytosolic mtDNA accumulation, suppressed cyclic GMP–AMP synthase–stimulator of interferon genes (cGAS–STING) signaling, and alleviated reperfusion injury (85). These results indicate that cardiomyocyte mitophagy can modify the post-ischemic immune microenvironment. However, because macrophage states were not directly traced, the study provides indirect rather than definitive evidence of cardiomyocyte–macrophage crosstalk. Endothelial mitophagy may also influence inflammatory-cell recruitment. Cai and colleagues (86) showed in cardiac microvascular endothelial cells and knockout mice that empagliflozin restored FUNDC1-dependent mitophagy, improved endothelial barrier integrity and microvascular perfusion, and reduced neutrophil infiltration. Experimental studies further indicate that defective mitochondrial quality control in macrophages is associated with persistent inflammatory activation, delayed efferocytosis, prolonged neutrophil accumulation, and adverse post-infarction remodeling (87). Conversely, restoration of mitochondrial homeostasis may support reparative macrophage functions, coordinated fibroblast activation, collagen deposition, angiogenesis, and scar maturation (88).

Macrophage origin and tissue localization are also important determinants of these effects. By integrating single-cell multi-omics, spatial analysis, and cell-depletion experiments, Xu and colleagues (89) identified a transient resident-macrophage state after MI that restrained excessive myofibroblast activation. This observation suggests that beneficial macrophage functions cannot be explained by a generalized increase in “M2 macrophages.” Instead, they depend on macrophage ontogeny, disease stage, and the local tissue niche.

**Figure 2:**
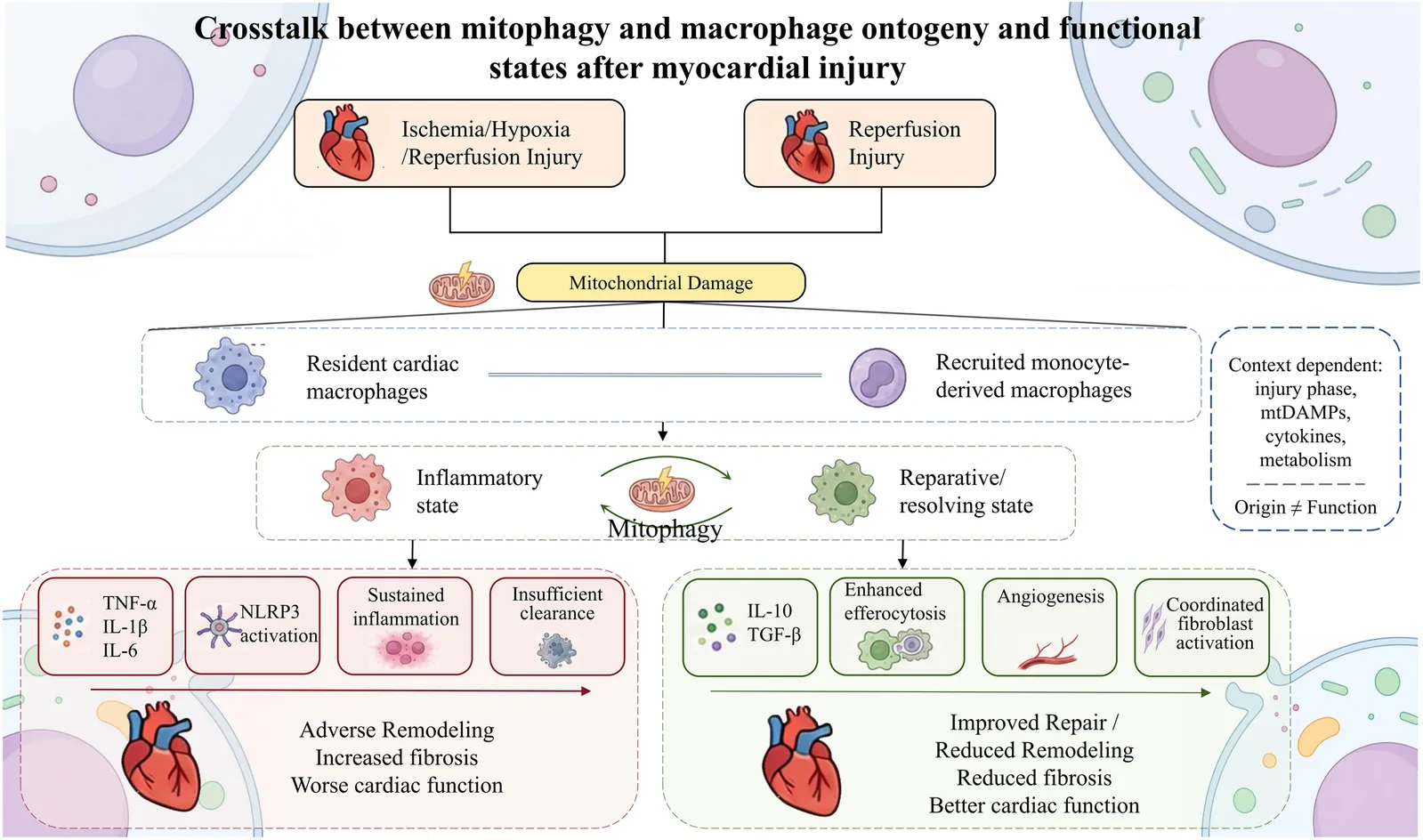
Crosstalk between mitophagy and macrophage ontogeny and functional states after myocardial injury.

The nature of mitophagy–macrophage crosstalk also differs across CVDs. In myocardial I/R injury, cardiomyocytes and endothelial cells rapidly release mitochondrial damage-associated signals, which promote acute sterile inflammation. In atherosclerosis, macrophages are exposed chronically to oxidized lipids, cholesterol accumulation, and defective mitochondrial clearance. Foam-cell formation, impaired mitophagy, and persistent inflammatory signaling therefore form a long-term feedback loop. Evidence that PARKIN-mediated mitophagy limits mtDNA accumulation and cGAS–STING activation further supports the connection between mitochondrial quality control and immune regulation (85). Overall, mitophagy and macrophage functional reprogramming constitute a bidirectional network centered on mitochondrial damage, innate immune signaling, and metabolic adaptation (90). Their effects are context dependent and should not be interpreted through a simple linear M1-to-M2 model. Further details are provided in Figure 2.

### 4.3 Therapeutic potential of interventions targeting mitophagy–macrophage polarization crosstalk in CVD

The translational potential of targeting the mitochondrial autophagy–macrophage crosstalk network cannot be evaluated simply by determining whether an intervention increases autophagy-related proteins or alters M1/M2-associated markers. Instead, three critical questions should be addressed. First, have randomized clinical trials demonstrated improvements in cardiovascular outcomes? Second, have corresponding alterations in target cells or inflammatory phenotypes been observed in human samples? Third, has the proposed mechanism been validated through genetic manipulation or rescue experiments demonstrating a causal role for mitochondrial autophagy–macrophage crosstalk? Based on these criteria, current interventions can be broadly classified into three categories: those with clinical cardiovascular benefits but unconfirmed crosstalk mechanisms, those with direct mechanistic evidence but limited to preclinical validation, and emerging strategies involving mitochondrial modulation or targeted delivery.

Sodium-glucose cotransporter 2 (SGLT2) inhibitors and glucagon-like peptide-1 receptor (GLP-1R) agonists represent therapeutic classes with the strongest clinical evidence. Large randomized clinical trials, including EMPEROR-Reduced and DELIVER, have demonstrated that SGLT2 inhibitors reduce the risk of heart failure hospitalization and cardiovascular death in patients with heart failure across a broad range of left ventricular ejection fractions (91, 92). Preclinical studies have further suggested that empagliflozin restores FUNDC1-dependent mitochondrial autophagy in cardiac microvascular endothelial cells and improves microvascular function (86). In a randomized study of 66 patients with MI and type 2 diabetes, Cliff and colleagues (93) compared early versus delayed empagliflozin initiation. Using macrophages differentiated from patient-derived monocytes, they observed alterations in inflammasome-related responses and caspase-1 activity after *ex vivo* stimulation. This study provided evidence linking clinical drug exposure to human macrophage responses. However, because mitochondrial autophagic flux was not directly assessed, it remains unclear whether the observed effects are mediated through mitochondrial autophagy–macrophage crosstalk. Similarly, semaglutide reduced major adverse cardiovascular events in the SELECT trial among adults with overweight or obesity and established cardiovascular disease (94). Experimental studies have also suggested that semaglutide can enhance PINK1/Parkin-dependent mitochondrial autophagy in hypoxia/reoxygenation-stressed cardiomyocytes (57). Nevertheless, direct evidence connecting these effects to human macrophage mitochondrial autophagy remains unavailable. Therefore, these therapies should be considered as agents with proven cardiovascular benefits and emerging mechanistic insights, rather than as clinically validated drugs that specifically target mitochondrial autophagy–macrophage crosstalk.

Direct interventions targeting components of this crosstalk network remain largely restricted to cellular and animal studies. Jin and colleagues (58) reported that the caspase-1 inhibitor VX765 prevents Parkin cleavage, restores mitochondrial autophagy and mitochondrial quality control in macrophages, and attenuates atherosclerosis in mice. Duan and colleagues (49) used bone marrow-specific genetic deletion models to demonstrate a causal role of AIBP in regulating macrophage mitochondrial autophagy and inflammatory activation. Huang and colleagues (95) further suggested that inhibition of miR-21-3p may correct excessive mitochondrial autophagy in cardiomyocytes and reduce the reciprocal amplification between injured cardiomyocytes and pro-inflammatory macrophages. The major strength of these studies is their mechanistic depth, supported by approaches such as conditional knockout, co-culture systems, and rescue experiments. However, cardiovascular outcome trials are currently lacking. Moreover, the optimal direction of mitochondrial autophagy regulation may differ among tissues and disease stages. Therefore, these findings require careful validation before clinical translation.

Mitochondria-targeted antioxidants, natural compounds, and extracellular vesicle-based approaches represent additional exploratory strategies, but their translational evidence remains limited. MitoQ has been reported to enhance PINK1/Parkin-associated mitochondrial autophagy in diabetic rat models of MIRI (96). A small randomized crossover trial in healthy older adults also suggested improvements in vascular endothelial function after MitoQ supplementation (97). However, evidence regarding cardiovascular event reduction and macrophage-related mechanisms remains insufficient. Natural compounds, including resveratrol and curcumin, have demonstrated anti-inflammatory and reparative effects in experimental MI models (98, 99). Melatonin has also been reported to regulate mitochondrial quality control by promoting damaged mitochondrial clearance or limiting excessive mitochondrial autophagy in different disease models (100). Nevertheless, differences in dosage, pharmacokinetics, bioavailability, and experimental systems limit direct extrapolation to clinical applications. Engineered extracellular vesicles provide another potential strategy by enabling the delivery of proteins, RNAs, or mitochondrial regulatory molecules. Although animal studies indicate that these vesicles may improve mitochondrial quality control and reduce reperfusion injury (101), challenges including cargo heterogeneity, tissue targeting, manufacturing consistency, and long-term safety remain unresolved.

Future therapeutic development should move beyond nonspecific enhancement of autophagy or generalized promotion of M2-like macrophage phenotypes and instead focus on precision regulation of mitochondrial quality control according to cell type, disease stage, and autophagic flux. First, dynamic assays that directly evaluate mitochondrial degradation and autophagic flux should replace reliance on static measurements of autophagy-related proteins. Second, therapeutic effects should be evaluated separately in cardiomyocytes, endothelial cells, fibroblasts, resident macrophages, and monocyte-derived macrophages to avoid unintended consequences of systemic modulation. Third, clinical studies should incorporate pharmacodynamic assessments, including human immune-cell profiling, circulating mitochondrial damage-associated molecules, and cardiac imaging, to determine whether modulation of this crosstalk pathway contributes to clinical benefit. Ultimately, establishing a continuous evidence chain from molecular target engagement and cell-specific mechanisms to *in vivo* efficacy and cardiovascular outcomes will be essential for transforming mitochondrial autophagy–macrophage crosstalk from a mechanistic concept into a clinically actionable therapeutic strategy.

## 5 Conclusions and prospects

This review positions mitophagy as a central link connecting mitochondrial quality control with pyroptotic signaling and macrophage functional remodeling in cardiovascular diseases, particularly MI and MIRI. When mitophagic flux is insufficient, damaged mitochondria accumulate and release mitochondrial reactive oxygen species, mitochondrial DNA, and other danger-associated signals, thereby promoting inflammasome activation, gasdermin-mediated membrane damage, and inflammatory amplification. Pyroptosis may, in turn, aggravate mitochondrial dysfunction and impair mitochondrial clearance, forming a self-reinforcing cycle of mitochondrial injury and sterile inflammation. However, the biological effects of mitophagy are not uniformly protective. Excessive mitochondrial removal may also compromise cellular energy metabolism and contribute to tissue injury. Therefore, cardiovascular protection depends on maintaining an appropriate and complete mitophagic flux rather than simply increasing mitophagy activity.

The relationship between mitophagy and macrophage function is similarly context dependent. Mitochondrial quality control influences macrophage redox balance, lysosomal function, efferocytosis, and metabolic adaptation, thereby shaping inflammatory or reparative functions. Nevertheless, this process cannot be adequately described as a linear transition from M1 to M2 macrophages. Macrophage populations in the injured heart differ in developmental origin, tissue localization, metabolic state, and functional properties, and several inflammatory and reparative states may coexist during the same disease phase. Moreover, mitophagy in cardiomyocytes, endothelial cells, and fibroblasts may indirectly regulate macrophage responses by altering the release of mitochondrial danger signals, vascular permeability, extracellular matrix remodeling, and the local inflammatory milieu. Conversely, macrophage-derived cytokines and metabolic mediators can feed back on mitochondrial homeostasis in neighboring cardiovascular cells. These observations support a multicellular and bidirectional crosstalk model rather than three independent pathological processes.

Despite increasing mechanistic evidence, several limitations should be recognized. First, most studies have been conducted in cultured cells and animal models, whereas direct evidence from human myocardial tissue, vascular specimens, and longitudinal clinical cohorts remains limited. Second, the consequences of mitophagy and pyroptosis vary according to cell type, disease stage, and the intensity and duration of stress. Static measurements of autophagy-related proteins are therefore insufficient to determine whether mitochondrial degradation is effectively completed. Third, interventions discussed in this review are supported by markedly different levels of evidence. SGLT2 and glucagon-like peptide-1 receptor agonists have demonstrated cardiovascular benefits in randomized clinical trials, but these benefits cannot yet be attributed specifically to modulation of mitophagy–pyroptosis–macrophage crosstalk. By contrast, caspase-1 inhibitors, mitochondrial regulatory molecules, natural compounds, and engineered extracellular vesicles have shown mechanistic potential mainly in preclinical models. Their target engagement, optimal dosage, tissue specificity, and long-term safety require further validation.

Future studies should therefore establish a continuous evidence chain from molecular mechanisms to clinical outcomes. Human tissue analysis should be integrated with single-cell sequencing, spatial transcriptomics, immune-cell profiling, and longitudinal cardiac imaging to define how this crosstalk network changes during the inflammatory, clearance, and reparative phases of cardiovascular injury. Cell-specific genetic models and dynamic mitophagy reporters are needed to distinguish the contributions of cardiomyocytes, endothelial cells, fibroblasts, resident cardiac macrophages, and recruited monocyte-derived macrophages. In parallel, pharmacological studies should determine the therapeutic window within which mitophagic flux can be restored without inducing excessive mitochondrial depletion. Clinical investigations should incorporate pharmacodynamic indicators, such as circulating mitochondrial damage-associated molecules, inflammasome activity, macrophage functional states, and direct measurements of mitochondrial quality control.

Overall, the therapeutic objective should not be the nonspecific enhancement of mitophagy, complete suppression of pyroptosis, or generalized induction of an M2-like phenotype. A more rational strategy is to restore appropriate mitochondrial quality control in specific cell populations, limit pathological inflammatory amplification, and preserve macrophage functions required for debris clearance, inflammation resolution, vascular repair, and scar maturation. Achieving this stage- and cell-specific regulation may enable the mitophagy–pyroptosis–macrophage crosstalk network to progress from a mechanistic framework toward a clinically actionable target for precision cardiovascular therapy.

## References

[1] Mensah GA Fuster V Murray CJL Roth GA Mensah GA Abate YH. Global burden of cardiovascular diseases and risks, 1990–2022. J Am Coll Cardiol. (2023) 82:2350–473. 10.1016/j.jacc.2023.11.007 38092509 PMC7615984

[2] Ajoolabady A Chiong M Lavandero S Klionsky DJ Ren J. Mitophagy in cardiovascular diseases: molecular mechanisms, pathogenesis, and treatment. Trends Mol Med. (2022) 28:836–49. 10.1016/j.molmed.2022.06.007 35879138 PMC9509460

[3] Libby P. Inflammation in atherosclerosis. Nature. (2002) 420:868–74. 10.1038/nature01323 12490960

[4] Tall AR Yvan-Charvet L. Cholesterol, inflammation and innate immunity. Nat Rev Immunol. (2015) 15:104–16. 10.1038/nri3793 25614320 PMC4669071

[5] Lu Y Li Z Zhang S Zhang T Liu Y Zhang L. Cellular mitophagy: mechanism, roles in diseases and small molecule pharmacological regulation. Theranostics. (2023) 13:736–66. 10.7150/thno.79876 36632220 PMC9830443

[6] Titus AS Sung E Zablocki D Sadoshima J. Mitophagy for cardioprotection. Basic Res Cardiol. (2023) 118:42. 10.1007/s00395-023-01009-x 37798455 PMC10556134

[7] Li A Gao M Liu B Qin Y Chen L Liu H. Mitochondrial autophagy: molecular mechanisms and implications for cardiovascular disease. Cell Death Dis. (2022) 13:444. 10.1038/s41419-022-04906-6 35534453 PMC9085840

[8] Liu P Wu H Ren H Wang J Yang F. Mastering cardiomyocyte mitophagy: molecular governance, pathological derailment and therapeutics. Peer J. (2026) 14:e20700. 10.7717/peerj.20700 41695696 PMC12903907

[9] O'Neill LAJ Kishton RJ Rathmell J. A guide to immunometabolism for immunologists. Nat Rev Immunol. (2016) 16:553–65. 10.1038/nri.2016.70 27396447 PMC5001910

[10] Frangogiannis NG. The inflammatory response in myocardial injury, repair, and remodeling. Nat Rev Cardiol. (2014) 11:255–65. 10.1038/nrcardio.2014.28 24663091 PMC4407144

[11] Liu D Qin H Gao Y Sun M Wang M. Cardiovascular disease: mitochondrial dynamics and mitophagy crosstalk mechanisms with novel programmed cell death and macrophage polarisation. Pharmacol Res. (2024) 206:107258. 10.1016/j.phrs.2024.107258 38909638

[12] Li M Zhang Y Hu Y Qin Y Zheng Y Lv S. Mitochondrial homeostasis meets novel programmed cell death: crosstalk mechanisms underlying cardiovascular diseases progression. Cell Commun Signal. (2026) 24:100. 10.1186/s12964-026-02673-x 41540474 PMC12888742

[13] Lemasters JJ. Selective mitochondrial autophagy, or mitophagy, as a targeted defense against oxidative stress, mitochondrial dysfunction, and aging. Rejuvenation Res. (2005) 8:3–5. 10.1089/rej.2005.8.3 15798367

[14] Zhao L Liu F Qi L Chen X Ning Y. Programmed cell death in allergic rhinitis: pathogenic mechanisms and therapeutic potential from a cellular perspective. Int Immunopharmacol. (2025) 164:115319. 10.1016/j.intimp.2025.115319 40773894

[15] Wang P Zhang N Wu B Wu S Zhang Y Sun Y. The role of mitochondria in vascular calcification. J Transl Int Med. (2020) 8:80–90. 10.2478/jtim-2020-0013 32983930 PMC7500121

[16] Bader V Winklhofer KF. PINK1 and Parkin: team players in stress-induced mitophagy. Biol Chem. (2020) 401:891–9. 10.1515/hsz-2020-0135 32297878

[17] Han R Liu Y Li S Li X Yang W. PINK1-PRKN mediated mitophagy: differences between *in vitro* and *in vivo* models. Autophagy. (2023) 19:1396–405. 10.1080/15548627.2022.2139080 36282767 PMC10240983

[18] Narendra DP Youle RJ. The role of PINK1-Parkin in mitochondrial quality control. Nat Cell Biol. (2024) 26:1639–51. 10.1038/s41556-024-01513-9 39358449

[19] Liu F Zhao L Wu T Yu W Li J Wang W. Targeting autophagy with natural products as a potential therapeutic approach for diabetic microangiopathy. Front Pharmacol. (2024) 15:1364616. 10.3389/fphar.2024.1364616 38659578 PMC11039818

[20] Dong Y Zhang X. Targeting cellular mitophagy as a strategy for human cancers. Front Cell Dev Biol. (2024) 12:1431968. 10.3389/fcell.2024.1431968 39035027 PMC11257920

[21] Lv Y Yu Z Zhang P Zhang X Li H Liang T. The structure and function of FUN14 domain-containing protein 1 and its contribution to cardioprotection by mediating mitophagy. Front Pharmacol. (2024) 15:1389953. 10.3389/fphar.2024.1389953 38828457 PMC11140143

[22] Gao A Jiang J Xie F Chen L. BNIP3 in mitophagy: novel insights and potential therapeutic target for diseases of secondary mitochondrial dysfunction. Clin Chim Acta. (2020) 506:72–83. 10.1016/j.cca.2020.02.024 32092316

[23] Yoo S-M Yamashita S- Kim H Na DH Lee H Kim SJ. FKBP8 LIRL-dependent mitochondrial fragmentation facilitates mitophagy under stress conditions. FASEB J. (2020) 34:2944–57. 10.1096/fj.201901735R 31908024

[24] Tang M Outissint I Chen Y Gong X. Comprehensive insights into mitophagy: mechanisms, disease associations, and therapeutic implications. J Cell Biochem. (2025) 126:e70056. 10.1002/jcb.70056 40714832

[25] Yamaguchi O Murakawa T Nishida K Otsu K. Receptor-mediated mitophagy. J Mol Cell Cardiol. (2016) 95:50–6. 10.1016/j.yjmcc.2016.03.010 27021519

[26] Bhujabal Z Birgisdottir ÅB Sjøttem E Brenne HB Øvervatn A Habisov S. FKBP8 recruits LC3A to mediate Parkin-independent mitophagy. EMBO Rep. (2017) 18:947–61. 10.15252/embr.201643147 28381481 PMC5452039

[27] Zhao X Wang Z Wang L Jiang T Dong D Sun M. The PINK1/Parkin signaling pathway-mediated mitophagy: a forgotten protagonist in myocardial ischemia/reperfusion injury. Pharmacol Res. (2024) 209:107466. 10.1016/j.phrs.2024.107466 39419133

[28] Liu H Zhao S Gao H Wang Y Gao J Guo P. Mitochondrial homeostasis: the central hub governing the progression of atherosclerosis. Precis Clin Med. (2026) 9:pbag10. 10.1093/pcmedi/pbag010 PMC13070710 41978695

[29] Shires SE Gustafsson ÅB. Mitophagy and heart failure. J Mol Med (Berl.). (2015) 93:253–62. 10.1007/s00109-015-1254-6 25609139 PMC4334711

[30] Luan Y Luan Y Feng Q Chen X Ren K Yang Y. Emerging role of mitophagy in the heart: therapeutic potentials to modulate mitophagy in cardiac diseases. Oxid Med Cell Longev. (2021) 2021:3259963. 10.1155/2021/3259963 34603595 PMC8483925

[31] Kayagaki N Stowe IB Lee BL O’Rourke K Anderson K Warming S. Caspase-11 cleaves gasdermin D for non-canonical inflammasome signaling. Nature. (2015) 526:666–71. 10.1038/nature15541 26375259

[32] Su K Shen C Wu W Zhou F Ye H. Post-translational modifications of gasdermin D: implications for pyroptosis and autoimmune diseases. Int Immunopharmacol. (2026) 179:116605. 10.1016/j.intimp.2026.116605 41946127

[33] Chen G Zhang Z Chong W Qiu G Li Z Yang S. The molecular mechanisms of pyroptosis and its implications in tumor immunotherapy. Mol Cancer. (2026) 25:63. 10.1186/s12943-026-02569-x 41606590 PMC12973759

[34] Liu F Yang Z Li J Wu T Li X Zhao L. Targeting programmed cell death in diabetic kidney disease: from molecular mechanisms to pharmacotherapy. Mol Med. (2024) 30:265. 10.1186/s10020-024-01020-5 39707216 PMC11660506

[35] Liu X Li Z Wang S Qiao Q Ye X Cheng L. Pyroptosis in cardiovascular diseases: molecular mechanisms, pathological roles, and therapeutic implications. Am J Cardiovasc Dis. (2025) 15:477–508. 10.62347/MTTP3167 41567846 PMC12816780

[36] van Hout GPJ Bosch L Ellenbroek GHJM de Haan JJ van Solinge WW Cooper MA. The selective NLRP3-inflammasome inhibitor MCC950 reduces infarct size and preserves cardiac function in a pig model of myocardial infarction. Eur Heart J. (2017) 38:828–36. 10.1093/eurheartj/ehw247 27432019

[37] Ding J Wang K Liu W She Y Sun Q Shi J. Pore-forming activity and structural autoinhibition of the gasdermin family. Nature. (2016) 535:111–6. 10.1038/nature18590 27281216

[38] Broz P Dixit VM. Inflammasomes: mechanism of assembly, regulation and signaling. Nat Rev Immunol. (2016) 16:407–20. 10.1038/nri.2016.58 27291964

[39] Nakahira K Haspel JA Rathinam VAK Lee S-J Dolinay T Lam HC. Autophagy proteins regulate innate immune responses by inhibiting the release of mitochondrial DNA mediated by the NALP3 inflammasome. Nat Immunol. (2011) 12:222–30. 10.1038/ni.1980 21151103 PMC3079381

[40] Zhong Z Umemura A Sanchez-Lopez E Liang S Shalapour S Wong J. NF-*κ*B restricts inflammasome activation via elimination of damaged mitochondria. Cell. (2016) 164:896–910. 10.1016/j.cell.2015.12.057 26919428 PMC4769378

[41] Kim M Yoon J Ryu J. Mitophagy: a balance regulator of NLRP3 inflammasome activation. BMB Rep. (2016) 49:529–35. 10.5483/BMBRep.2016.49.10.115 27439607 PMC5227293

[42] Devant P Boršić E Ngwa EM Xiao H Chouchani ET Thiagarajah JR. Gasdermin D pore-forming activity is redox-sensitive. Cell Rep. (2023) 42:112008. 10.1016/j.celrep.2023.112008 36662620 PMC9947919

[43] Zhou R Yazdi AS Menu P Tschopp J. A role for mitochondria in NLRP3 inflammasome activation. Nature. (2011) 469:221–5. 10.1038/nature09663 21124315

[44] Gan Y-M Qin N-S Ouyang J Tang Y-M Qin J-H Wei S-M. Cross-regulation of autophagy and pyroptosis: a new perspective on the inflammatory microenvironment of atherosclerosis. Apoptosis. (2026) 31:18. 10.1007/s10495-025-02221-x 41518391

[45] Toldo S Mauro AG Cutter Z Abbate A. Inflammasome, pyroptosis, and cytokines in myocardial ischemia-reperfusion injury. Am J Physiol Heart Circ Physiol. (2018) 315:H1553–68. 10.1152/ajpheart.00158.2018 30168729 PMC6336966

[46] Chai R Xue W Shi S Zhou Y Du Y Li Y. Cardiac remodeling in heart failure: role of pyroptosis and its therapeutic implications. Front Cardiovasc Med. (2022) 9:870924. 10.3389/fcvm.2022.870924 35509275 PMC9058112

[47] Yang Y Zhu C Zhou X Zhang X. USP30 knockdown drives mitophagy and suppresses pyroptosis in heart failure by activating the PINK1/Parkin pathway. Int Heart J. (2025) 66:1002–14. 10.1536/ihj.24-738 41320325

[48] Duan Y Li Q Wu J Zhou C Liu X Yue J. A detrimental role of endothelial S1PR2 in cardiac ischemia-reperfusion injury via modulating mitochondrial dysfunction, NLRP3 inflammasome activation, and pyroptosis. Redox Biol. (2024) 75:103244. 10.1016/j.redox.2024.103244 38909407 PMC11254837

[49] Duan M Chen H Yin L Zhu X Novák P Lv Y. Mitochondrial apolipoprotein A-I binding protein alleviates atherosclerosis by regulating mitophagy and macrophage polarization. Cell Commun Signal. (2022) 20:60. 10.1186/s12964-022-00858-8 35525979 PMC9077873

[50] Wu B Xiang H Yu Y Liu S Song D Wu C. 3,4-Benzo[a]pyrene aggravates myocardial infarction injury by activating NLRP3-related pyroptosis through PINK1/Parkin-mitophagy-MPTP opening axis. Int Immunopharmacol. (2023) 122:110481. 10.1016/j.intimp.2023.110481 37390647

[51] Hu X He X Xiang M Wang Y Ma S Li M. TREM1 as a master regulator of mitophagy-pyroptosis crosstalk in myocardial infarction: a dual-target strategy for precision therapy. Int Immunopharmacol. (2026) 174:116353. 10.1016/j.intimp.2026.116353 41679181

[52] Peng X Chen H Li Y Huang D Huang B Sun D. Effects of NIX-mediated mitophagy on ox-LDL-induced macrophage pyroptosis in atherosclerosis. Cell Biol Int. (2020) 44:1481–90. 10.1002/cbin.11343 32181963

[53] Yin Z Jia X Zhang B Gong Y Zheng Q Lu L. SIRT3-BNIP3 signaling axis alleviates myocardial ischemia/reperfusion-induced cardiac injury via regulating GSDME. Cell Signal. (2026) 138:112202. 10.1016/j.cellsig.2025.112202 41173379

[54] Huang C Zhang X Wu S Chang Q Zheng Z Xu J. METTL3, m6a modification, and EGR1: interplay affecting myocardial I/R injury outcomes. Cell Biol Toxicol. (2024) 41:7. 10.1007/s10565-024-09937-7 39707117 PMC11662061

[55] Chen Y Yuan C Qin W Yu B Wei D Wu P. TMAO promotes vascular endothelial cell pyroptosis via the LPEAT-mitophagy pathway. Biochem Biophys Res Commun. (2024) 703:149667. 10.1016/j.bbrc.2024.149667 38382362

[56] Boshchenko AA Maslov LN Mukhomedzyanov AV Zhuravleva OA Slidnevskaya AS Naryzhnaya NV. Peptides are cardioprotective drugs of the future: the receptor and signaling mechanisms of the cardioprotective effect of glucagon-like peptide-1 receptor agonists. Int J Mol Sci. (2024) 25:4900. 10.3390/ijms25094900 38732142 PMC11084666

[57] Li L Jin L Tian Y Wang J. Semaglutide enhances PINK1/Parkin-dependent mitophagy in hypoxia/reoxygenation-induced cardiomyocyte injury. Mol Med Rep. (2025) 31:1–12. 10.3892/mmr.2025.13476 40017118 PMC11884227

[58] Jin Y Liu Y Xu L Xu J Xiong Y Peng Y. Novel role for caspase 1 inhibitor VX765 in suppressing NLRP3 inflammasome assembly and atherosclerosis via promoting mitophagy and efferocytosis. Cell Death Dis. (2022) 13:512. 10.1038/s41419-022-04966-8 35641492 PMC9156694

[59] Luo G Chen L Chen M Mao L Zeng Q Zou Y. Hirudin inhibit the formation of NLRP3 inflammasome in cardiomyocytes via suppressing oxidative stress and activating mitophagy. Heliyon. (2024) 10:e23077. 10.1016/j.heliyon.2023.e23077 38163129 PMC10754874

[60] Liu K Wang H Wang Y Zhang X Wang R Zhang Z. Exploring the therapeutic potential of SIRT6-enriched adipose stem cell-derived exosomes in myocardial ischemia-reperfusion injury: unfolding new epigenetic frontiers. Clin Epigenetics. (2024) 16:7. 10.1186/s13148-023-01618-2 38172884 PMC10765803

[61] Tauber AI. Metchnikoff and the phagocytosis theory. Nat Rev Mol Cell Biol. (2003) 4:897–901. 10.1038/nrm1244 14625539

[62] Arango DG Descoteaux A. Macrophage cytokines: involvement in immunity and infectious diseases. Front Immunol. (2014) 5:491. 10.3389/fimmu.2014.00491 25339958 PMC4188125

[63] Coillard A Segura E. *In vivo* differentiation of human monocytes. Front Immunol. (2019) 10:1907. 10.3389/fimmu.2019.01907 31456804 PMC6700358

[64] Bertrand JY Jalil A Klaine M Jung S Cumano A Godin I. Three pathways to mature macrophages in the early mouse yolk sac. Blood. (2005) 106:3004–11. 10.1182/blood-2005-02-0461 16020514

[65] Nobs SP Kopf M. Tissue-resident macrophages: guardians of organ homeostasis. Trends Immunol. (2021) 42:495–507. 10.1016/j.it.2021.04.007 33972166

[66] Park MD Silvin A Ginhoux F Merad M. Macrophages in health and disease. Cell. (2022) 185:4259–79. 10.1016/j.cell.2022.10.007 36368305 PMC9908006

[67] Shapouri-Moghaddam A Mohammadian S Vazini H Taghadosi M Esmaeili S-A Mardani F. Macrophage plasticity, polarization, and function in health and disease. J Cell Physiol. (2018) 233:6425–40. 10.1002/jcp.26429 29319160 PMC13482560

[68] Mantovani A Biswas SK Galdiero MR Sica A Locati M. Macrophage plasticity and polarization in tissue repair and remodeling. J Pathol. (2013) 229:176–85. 10.1002/path.4133 23096265

[69] Avila-Ponce DLU Vazquez-Jimenez A Matadamas-Guzman M Pelayo R Resendis-Antonio O. Transcriptional and microenvironmental landscape of macrophage transition in cancer: a boolean analysis. Front Immunol. (2021) 12:642842. 10.3389/fimmu.2021.642842 34177892 PMC8222808

[70] Kadl A Meher AK Sharma PR Lee MY Doran AC Johnstone SR. Identification of a novel macrophage phenotype that develops in response to atherogenic phospholipids via NRF2. Circ Res. (2010) 107:737–46. 10.1161/CIRCRESAHA.109.215715 20651288 PMC2941538

[71] Yang S Yuan H-Q Hao Y-M Ren Z Qu S-L Liu L-S. Macrophage polarization in atherosclerosis. Clin Chim Acta. (2020) 501:142–6. 10.1016/j.cca.2019.10.034 31730809

[72] Chazaud B. Macrophages: supportive cells for tissue repair and regeneration. Immunobiology. (2014) 219:172–8. 10.1016/j.imbio.2013.09.001 24080029

[73] Yang Z Lin S Feng W Liu Y Song Z Pan G. A potential therapeutic target in traditional Chinese medicine for ulcerative colitis: macrophage polarization. Front Pharmacol. (2022) 13:999179. 10.3389/fphar.2022.999179 36147340 PMC9486102

[74] Jung S-H Hwang B-H Shin S Park E-H Park S-H Kim CW. Spatiotemporal dynamics of macrophage heterogeneity and a potential function of TREM2(hi) macrophages in infarcted hearts. Nat Commun. (2022) 13:4580. 10.1038/s41467-022-32284-2 35933399 PMC9357004

[75] Rizzo G Gropper J Piollet M Vafadarnejad E Rizakou A Bandi SR. Dynamics of monocyte-derived macrophage diversity in experimental myocardial infarction. Cardiovasc Res. (2023) 119:772–85. 10.1093/cvr/cvac113 35950218 PMC10153424

[76] Deberge M Chaudhary R Schroth S Thorp EB. Immunometabolism at the heart of cardiovascular disease. JACC Basic Transl Sci. (2023) 8:884–904. 10.1016/j.jacbts.2022.12.010 37547069 PMC10401297

[77] Zhong Y Xu C Li J Liang Z Wang M Ma C. Mitochondrial dynamics and metabolism in macrophages for cardiovascular disease: a review. Phytomedicine. (2025) 140:156620. 10.1016/j.phymed.2025.156620 40068296

[78] Kong Y Zhang Q Wang S Li R Fu C Wei Q. Mitochondrial metabolism regulated macrophage phenotype in myocardial infarction. Biomed Pharmacother. (2024) 180:117494. 10.1016/j.biopha.2024.117494 39321509

[79] Wang K Wei W Wang J Cong Y Wang H An C. Protein kinase B is involved in bisphenol A-induced macrophage polarization through mechanistic target of rapamycin-dependent autophagy. J Transl Int Med. (2026) 14:485–87. 10.1515/jtim-2026-0029 42389431 PMC13320531

[80] Mouton AJ DeLeon-Pennell KY Rivera Gonzalez OJ Flynn ER Freeman TC Saucerman JJ. Mapping macrophage polarization over the myocardial infarction time continuum. Basic Res Cardiol. (2018) 113:26. 10.1007/s00395-018-0686-x 29868933 PMC5986831

[81] Choi S-H Agatisa-Boyle C Gonen A Kim A Kim J Alekseeva E. Intracellular AIBP (apolipoprotein A-I binding protein) regulates oxidized LDL (low-density lipoprotein)-induced mitophagy in macrophages. Arterioscler Thromb Vasc Biol. (2021) 41:e82–96. 10.1161/ATVBAHA.120.315485 33356389 PMC8105271

[82] Wu KKL Long KK Lin H Siu PMF Hoo RLC Ye D. The APPL1-RAB5 axis restricts NLRP3 inflammasome activation through early endosomal-dependent mitophagy in macrophages. Nat Commun. (2021) 12:6637. 10.1038/s41467-021-26987-1 34789781 PMC8599493

[83] Nicolás-Ávila JA Pena-Couso L Muñoz-Cánoves P Hidalgo A. Macrophages, metabolism and heterophagy in the heart. Circ Res. (2022) 130:418–31. 10.1161/CIRCRESAHA.121.319812 35113662

[84] Xu S Xu C Xu J Zhang K Zhang H. Macrophage heterogeneity and its impact on myocardial ischemia-reperfusion injury: an integrative review. J Inflamm Res. (2023) 16:5971–87. 10.2147/JIR.S436560 38088942 PMC10712254

[85] Li Y Wang Y Zhang H Xu P Yuan X Yuan H. Parkin overexpression confers cardioprotection via suppressing the mtDNA-cGAS-STING axis in myocardial ischemia/reperfusion injury. Basic Res Cardiol. (2026) 121:395–412. 10.1007/s00395-026-01169-6 41824029

[86] Cai C Guo Z Chang X Li Z Wu F He J. Empagliflozin attenuates cardiac microvascular ischemia/reperfusion through activating the AMPK*α*1/ULK1/FUNDC1/mitophagy pathway. Redox Biol. (2022) 52:102288. 10.1016/j.redox.2022.102288 35325804 PMC8938627

[87] Xu A Xu S Tan X Sun Q Song Y Nong Y. Dynamic regulation of macrophage polarization in acute myocardial infarction and its therapeutic potential. J Inflamm Res. (2025) 18:17363–85. 10.2147/JIR.S543139 41416126 PMC12711377

[88] Yap J Irei J Lozano-Gerona J Vanapruks S Bishop T Boisvert WA. Macrophages in cardiac remodeling after myocardial infarction. Nat Rev Cardiol. (2023) 20:373–85. 10.1038/s41569-022-00823-5 36627513

[89] Xu Y Jiang K Su F Deng R Cheng Z Wang D. A transient wave of BHLHE41(+) resident macrophages enables remodeling of the developing infarcted myocardium. Cell Rep. (2023) 42:113174. 10.1016/j.celrep.2023.113174 37751357

[90] Li Z Yan N Pei X Duan X Tian H Wang H. Paeonol attenuates atherosclerosis by inhibiting mTOR via AMPK and SIRT1: modulating lipid metabolism, autophagy, and pyroptosis. J Transl Int Med. (2026) 14:647–49. 10.1515/jtim-2026-0041 42657052 PMC13508827

[91] Packer M Anker SD Butler J Filippatos G Pocock SJ Carson P. Cardiovascular and renal outcomes with empagliflozin in heart failure. N Engl J Med. (2020) 383:1413–24. 10.1056/NEJMoa2022190 32865377

[92] Solomon SD McMurray JJV Claggett B De Boer RA DeMets D Hernandez AF. Dapagliflozin in heart failure with mildly reduced or preserved ejection fraction. N Engl J Med. (2022) 387:1089–98. 10.1056/NEJMoa2206286 36027570

[93] Cliff CL Shah MU Ward JK Inghels M Lee K Squires PE. Timing-dependent anti-inflammatory effects of empagliflozin in monocyte-derived macrophages from post-myocardial infarct patients with type 2 diabetes. Cardiovasc Diabetol. (2026) 25:43. 10.1186/s12933-025-03042-7 41680826 PMC12896262

[94] Lincoff AM Brown-Frandsen K Colhoun HM Deanfield J Emerson SS Esbjerg S. Semaglutide and cardiovascular outcomes in obesity without diabetes. N Engl J Med. (2023) 389:2221–32. 10.1056/NEJMoa2307563 37952131

[95] Huang Y Huang Y Cai Z Ferrari MW Li C Zhang T. MiR-21-3p inhibitor exerts myocardial protective effects by altering macrophage polarization state and reducing excessive mitophagy. Commun Biol. (2024) 7:1371. 10.1038/s42003-024-07050-3 39438580 PMC11496525

[96] Ji Y Leng Y Lei S Qiu Z Ming H Zhang Y. The mitochondria-targeted antioxidant MitoQ ameliorates myocardial ischemia-reperfusion injury by enhancing PINK1/Parkin-mediated mitophagy in type 2 diabetic rats. Cell Stress Chaperones. (2022) 27:353–67. 10.1007/s12192-022-01273-1 35426609 PMC9346044

[97] Rossman MJ Santos-Parker JR Steward CAC Bispham NZ Cuevas LM Rosenberg HL. Chronic supplementation with a mitochondrial antioxidant (MitoQ) improves vascular function in healthy older adults. Hypertension. (2018) 71:1056–63. 10.1161/HYPERTENSIONAHA.117.10787 29661838 PMC5945293

[98] Liu S Du Y Shi K Yang Y Yang Z. Resveratrol improves cardiac function by promoting M2-like polarization of macrophages in mice with myocardial infarction. Am J Transl Res. (2019) 11:5212–26. 31497235 PMC6731431

[99] Yan S Zhou M Zheng X Xing Y Dong J Yan M. Anti-inflammatory effect of curcumin on the mouse model of myocardial infarction through regulating macrophage polarization. Mediators Inflamm. (2021) 2021:9976912. 10.1155/2021/9976912 34462629 PMC8403049

[100] Wang Z Lin D Cui B Zhang D Wu J Ma J. Melatonin protects against myocardial ischemia-reperfusion injury by inhibiting excessive mitophagy through the apelin/SIRT3 signaling axis. Eur J Pharmacol. (2024) 963:176292. 10.1016/j.ejphar.2023.176292 38128867

[101] Jing Y Cai Y Li Q Zheng Z Yang H Yu Y. Empagliflozin-pretreated BMSC exosomes attenuate myocardial ischemia-reperfusion injury by enhancing ATAD3A/PINK1-dependent mitophagy. Stem Cell Res Ther. (2025) 16:595. 10.1186/s13287-025-04715-6 41163081 PMC12573846

